# Is it necessary for all patients with suspicious lesions undergo systematic biopsy in the era of MRI-TRUS fusion targeted biopsy?

**DOI:** 10.1590/S1677-5538.IBJU.2023.0060

**Published:** 2023-03-20

**Authors:** Zhengtong Lv, Jinfu Wang, Miao Wang, Huimin Hou, Liuqi Song, Haodong Li, Xuan Wang, Ming Liu

**Affiliations:** 1 National Center of Gerontology Institute of Geriatric Medicine Chinese Academy of Medical Sciences P.R China Department of Urology, Beijing Hospital, National Center of Gerontology; Institute of Geriatric Medicine, Chinese Academy of Medical Sciences, P.R. China;; 2 Graduate School of Peking Union Medical College Chinese Academy of Medical Sciences P.R China Graduate School of Peking Union Medical College and Chinese Academy of Medical Sciences, P.R. China;; 3 Peking University Fifth School of Clinical Medicine P.R China Peking University Fifth School of Clinical Medicine, P.R. China;; 4 Peking University China-Japan Friendship School of Clinical Medicine P.R China Peking University China-Japan Friendship School of Clinical Medicine, P.R. China

**Keywords:** Prostatic Neoplasms, Prostate, Magnetic Resonance Imaging

## Abstract

**Purpose:**

Targeted biopsy (TB) combined with systematic biopsy (SB) is an optimized mode of prostate biopsy but can often lead to oversampling and overdiagnosis accompanied by potential biopsy-related complications and patient discomfort. Here, we attempted to reasonably stratify the patient population based on multi-parameter indicators with the aim of avoiding unnecessary SB.

**Methods:**

In total, 340 biopsy-naïve men with suspected lesions, prostate-specific antigen (PSA) < 20 ng/mL and prostate imaging-reporting and data system (PI-RADS) ≥ 3 enrolled for study underwent both TB and SB. The primary outcome was to determine independent predictors for a valid diagnosis, assuming that only TB was performed and SB omitted (defined as mono-TB), taking TB + SB as the reference standard. The secondary outcomes were exploration of the predictive factors of mono-TB and TB + SB in detection of prostate cancer (PCa) and clinically significant PCa (csPCa).

**Results:**

The mean PSA density (PSAD) of patient group was 0.27 ng/mL/mL. Multiparametric MRI PI-RADS scores were 3-5 in 146 (42.94%), 105 (30.88%), and 89 (26.18%) cases, respectively. PCa and csPCa were detected in 178/340 (52.35%) and 162/340 (47.65%) patients, respectively. Overall, 116/178 (65.17%) patients diagnosed with PCa displayed pathological consistencies between mono-TB and TB + SB modes. PSAD and PI-RADS were independent predictors of valid diagnosis using mono-TB.

**Conclusions:**

PSAD combined with PI-RADS showed utility in guiding optimization of the prostate biopsy mode. Higher PSAD and PI-RADS values were associated with greater confidence in implementing mono-TB and safely omitting SB, thus effectively balancing the benefits and risks.

## INTRODUCTION

Over the past few years, multiparametric magnetic resonance imaging (MRI) has played an increasingly important role in the diagnosis of prostate cancer (PCa) (1). MRI images are superimposed with real-time transrectal ultrasonography (TRUS) images through cognition or software assistance for examining potential suspected tumor areas with the purpose of achieving targeted biopsy (TB) (2). Although supplementation with MRI has increased sensitivity in the detection of clinically significant PCa (csPCa) (3), omission of systematic biopsy (SB) for all patients is associated with risk of diagnosis failure in ~8.8% csPCa cases (4). Data from several large randomized controlled trials suggest that MRI-TRUS fusion-targeted biopsy combined with systematic biopsy (TB + SB) presents the optimal choice (4, 5).

While the TB + SB method significantly enhances detection of high-risk or csPCa (6), overdiagnosis of low-volume, low-risk, clinically insignificant PCa (cisPCa) with combined biopsy has also been reported (4, 7). In addition, increase in the number of biopsy cores leads to greater patient discomfort and risk of infection and bleeding (8, 9). Furthermore, for patients diagnosed with PCa that need follow-up surgery, tissue adhesion caused by multi-needle biopsy may increase the difficulty of surgery, along with the probability of intraoperative and postoperative complications (10, 11).

Accordingly, we propose that the fixed TB+SB mode is not required for all patients and the patient population only requiring TB can be screened based on clinical indicators, particularly in the current era of precise MRI-TRUS fusion-guided biopsy. The purpose of this study was to distinguish the subset of patients suitable for TB only through evaluation of indicators of clinical characteristics without missing diagnosis or overdiagnosis of PCa.

## MATERIALS AND METHODS

### Study design

We recruited patients who received MRI-TRUS fusion TB + SB in Beijing hospital from January 2018 to September 2022 as part of an ongoing prospective trial, with approval from the Ethics Committee of Beijing Hospital (2018BJYYEC-028-02), registered in the Chinese clinical trial registry (ChiCTR1800018575). Using known pathological results of TB + SB as the gold standard, all patients were self-controlled to assess the pathological outcome under the premise of receiving only TB and omitting SB (defined as mono-TB).

### Study population

**Figure 1:**
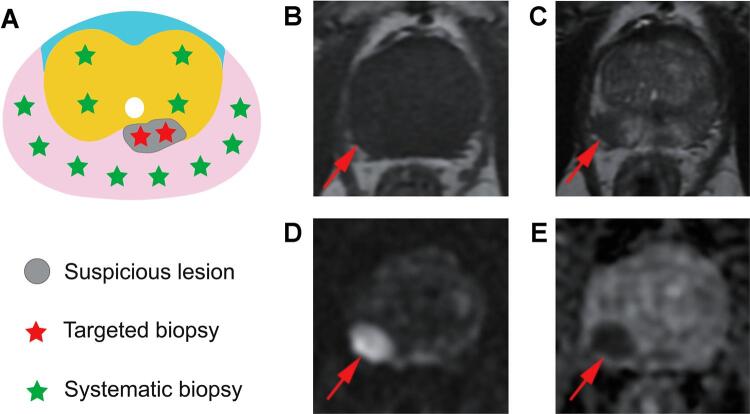
Biopsy mode diagram and example of mpMRI images. (A) TB/SB mode and nine regions of prostate. (B-E) A PI-RADS score 4 lesion in the peripheral zone of the right prostate. No obvious signal abnormality on T1WI, hypointense signal on T2WI, hyperintense signal on DWI and hypointense signal on ADC.

Inclusion criteria were as follows: patients with suspected PCa who underwent MRI-TRUS fusion TB + SB (Figure-1A), prostate-specific antigen (PSA) < 20 ng/mL, Prostate Imaging Reporting & Data System (PI-RADS) score ≥ 3, age < 75 years, prostate biopsy naïve, no exposure to androgen deprivation therapy, radiotherapy, and chemotherapy, and with informed consent. Exclusion criteria included previous diagnosis of PCa, previous prostate surgery or prostate biopsy, and no provision of signed informed consent.

mpMRI = multiparametric magnetic resonance imaging; TB = Targeted biopsy; SB = Systematic biopsy; PI-RADS = Prostate imaging-reporting and data system; T1WI = T1-weighted image; T2WI = T2-weighted image; DWI = Diffusion-weighted imaging; ADC = Apparent diffusion coefficient.

### Imaging and biopsy process

Clinicopathological data of all patients were collected, including age, digital rectal examination (DRE), PSA, prostate volume, PSA density (PSAD), MRI information and pathological results. All patients underwent MRI using a 3.0T MR scanner (MAGNETOM Prisma™, Siemens Healthcare, Erlangen, Germany) equipped with an 18-channel cardiac phased-array coil. MRI protocols included axial T1-weighted imaging, triaxial (axial, sagittal and coronal) T2-weighted imaging, diffusion-weighted imaging, and apparent diffusion coefficient. (Supplementary Table-1; Figures 1B-E). All suspicious lesions were classified according to the guidelines of PI-RADS version 2.1. In cases where multiple lesions were identified, the highest PI-RADS score was taken as the primary score. All MRI images were analyzed by two senior radiologists without any clinical information. The location, diameter and number of suspicious lesions were recorded. In the case of any disagreements in PI-RADS scoring, a consensus was reached through negotiation.

### Biopsy process

In each patient, at least two but no more than four cores were cognitive-targeted for each suspected lesion of the prostate in the MRI-TRUS fusion image by one urologist, followed by at least one core per zone via the systematic perineal approach by another urologist (Figure-1A). Both urologists had more than two years of experience in prostate biopsy, and MRI data were unknown to SB performers. All biopsy specimens were examined pathologically by two experienced pathologists without any clinical information.

### Definitions

csPCa was defined as any Gleason score ≥ 3 + 4 (ISUP grade ≥ 2) (12). Cases where the pathology determined with TB + SB was PCa but that with mono-TB was not PCa were defined as missed detection. Cases where the results of mono-TB were downgraded from csPCa to cisPCa were defined as risk stratification misjudgment. Valid diagnosis was defined in cases where pathological results were consistent between mono-TB and TB + SB modes. Otherwise, the missed detection and risk stratification misjudgment mentioned above were classified as invalid diagnosis.

## Statistical Analysis

SPSS Version 23.0 (IBM Corp., Armonk, NY, USA) statistical software was used for data processing. Continuous variables were expressed as means ± standard deviation (SD). Frequencies and proportions were reported for classification variables. Univariate and multivariate logistic regression analyses (Method: Enter) were applied to obtain predictors of valid diagnosis of mono-TB. The ROC curve was used to evaluate the predictive value. The weighted kappa test was employed to assess the consistency in results between TB and TB+SB modes. Differences were considered statistically significant at P < 0.05.

## RESULTS

### Study population

**Figure 2:**
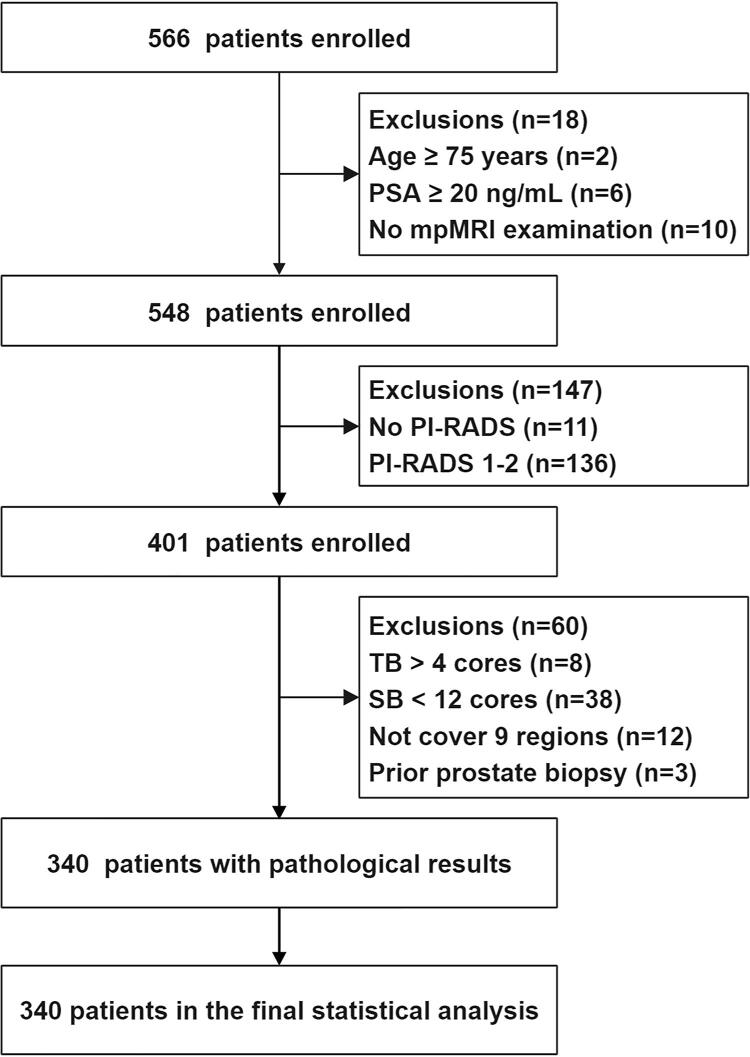
Study cohort flow diagram.

In total, 340 patients were included in the final analysis (Figure-2). Basic clinical information of patients is presented in Table-1. The average patient age was 64.88 years and average PSA level was 8.23. The average numbers of TB and SB cores per patient were 4.68 and 16.41, respectively. Among the 340 participants, 175 (51.47%) had a positive digital rectal examination (DRE). The MRI PI-RADS scores were 3, 4, and 5 in 146 (42.94%), 105 (30.88%), and 89 (26.18%) cases, respectively.

**Table 1:** Patients characteristics.

Variable	Descriptive statistics	Value
Number of patients	N	340
Age (Years)	means ± SD	64.88 ± 5.63
PSA (ng/mL)	means ± SD	8.23 ± 4.28
Prostate volume (mL)	means ± SD	39.25 ± 20.74
PSAD (ng/mL/mL)	means ± SD	0.27 ± 0.23
Total cores	means ± SD	21.09 ± 3.27
TB cores	means ± SD	4.68 ± 2.04
**DRE**		
Negtive	n (%)	165 (48.53%)
Positive	n (%)	175 (51.47%)
Lesions number	means ± SD	2.14 ± 1.04
Lesion size (cm)	means ± SD	1.43 ± 0.46
**Lesion location**		
Peripheral zone	n (%)	161 (47.35%)
Transitional zone	n (%)	99 (29.12%)
Both	n (%)	80 (23.53%)
**mpMRI**		
PI-RADS 3	n (%)	146 (42.94%)
PI-RADS 4	n (%)	105 (30.88%)
PI-RADS 5	n (%)	89 (26.18%)

PSA = Prostate-specific antigen; PSAD = Prostate-specific antigen density; TB = Targeted biopsy; DRE = Digital rectal examination; mpMRI = multiparametric magnetic resonance imaging; PI-RADS = Prostate imaging-reporting and data system; SD = Standard deviation.

### Biopsy outcomes of TB + SB and mono-TB

Results from the two biopsy modes are presented in Table-2. In the TB + SB mode, 178 (52.35%) individuals were diagnosed with PCa, including 140 (41.18%) csPCa and 38 (11.18%) cisPCa. In the mono-TB mode, the detection rate was lower for PCa and csPCa, but higher for cisPCa. A similar trend was observed in the pathology Gleason score, where the proportion of patients with Gleason 6 was increased with the mono-TB mode and the proportion with Gleason 7-10 decreased, compared with the TB + SB mode, although data were not statistically significant (P > 0.05).

**Table 2:** Biopsy outcomes by Chi-square test.

Outcome	TB + SB	TB	P-value
**Cancer detection**			0.05
No PCa	162 (47.65%)	178 (52.35%)	
csPCa	140 (41.18%)	111 (32.65%)	
cisPCa	38 (11.18%)	51 (15.00%)	
**Gleason score**			0.27
Gleason 6	38 (11.18%)	51 (15.00%)	
Gleason 7	107 (31.47%)	88 (25.88%)	
Gleason 8	18 (5.29%)	12 (3.53%)	
Gleason 9	10 (2.94%)	7 (2.06%)	
Gleason 10	5 (1.47%)	4 (1.18%)	

TB = Targeted biopsy; SB = Systematic biopsy; PCa = Prostate cancer; csPCa = clinically significant prostate cancer; cisPCa = clinically insignificant prostate cancer.

Univariate and multivariate logistic regression analyses were performed to explore the predictive factors of these two biopsy modes in detection of PCa and csPCa. In the TB + SB mode, age and PI-RADS were significant predictors for PCa and PSAD and PI-RADS for csPCa detection (Supplementary Table-2). In the mono-TB mode, PSAD and PI-RADS were significant predictors for PCa and age, DRE, PSAD, and PI-RADS for csPCa detection (Supplementary Table-3).

### Validity analysis of mono-TB

**Figure 3:**
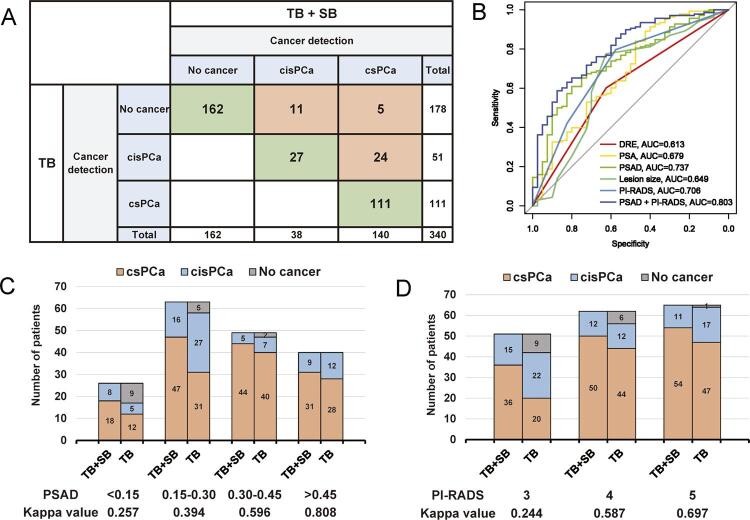
Validity analysis of mono-TB. (A) Comparison of pathology between mono-TB and TB + SB modes for benign/csPCa/cisPCa. (B) ROC curve analysis of each factor in predicting validity of diagnosis of mono-TB. (C, D) Pathological differences between mono-TB and TB + SB modes for benign/csPCa/cisPCa detection according to PSAD and PI-RADS levels.

Among the 178 patients diagnosed with PCa, the valid diagnosis rate of mono-TB was 77.53%. Overall, detection of benign/csPCa/cisPCa was consistent in 138 patients, regardless of whether TB + SB or mono-TB was used. The details of missed detection and risk stratification misjudgment are shown in Figure-3A. Invalid diagnosis was mainly caused by misdiagnosis of csPCa as cisPCa.

TB = Targeted biopsy; SB = Systematic biopsy; csPCa = clinically significant prostate cancer; cisPCa = clinically insignificant prostate cancer; ROC = Receiver operator characteristic.

Univariate and multivariate logistic analyses were conducted to confirm the significant predictors of valid diagnosis in the mono-TB mode. PI-RADS and PSAD were consistently identified as independent predictors (Table-3). ROC curve analysis revealed that the AUC values of PSAD and PI-RADS were higher than other indexes in predicting valid diagnosis in the mono-TB mode. Upon combination of PSAD and PI-RADS, the AUC value increased to 0.803 (Figure-3B). The optimal threshold sensitivity was 0.587 while specificity was up to 0.875.

**Table 3:** Univariate and multivariate logistic regression analyses to predict validity for TB.

		Univariate analysis		Multivariate analysis	
Variable		OR (95% CI)	P-value	OR (95% CI)	P-value
Age (Years)		1.006 (0.939-1.079)	0.856		
DRE		2.515 (1.218-5.194)	0.013	2.016 (0.899-4.523)	0.089
PSA (ng/mL)		1.188 (1.074-1.314)	0.001	1.019 (0.901-1.153)	0.762
Prostate-Vol (mL)		0.985 (0.967-1.004)	0.127		
PSAD (ng/mL2)		386.9 (16.62-8189)	0.001	151.7 (4.674-4924)	0.005
Lesions number		0.756 (0.535-1.068)	0.113		
Lesion size (cm)		3.055 (1.343-6.947)	0.008	0.830 (0.232-2.975)	0.775
Lesion location		1.543 (0.927-2.567)	0.095		
PI-RADS		2.797 (1.703-4.596)	0.001	2.663 (1.195-5.936)	0.017

TB = Targeted biopsy; DRE = Digital rectal examination; PSA = Prostate-specific antigen; PSAD = Prostate-specific antigen density; PI-RADS = Prostate imaging-reporting and data system; OR = Odds ratio; CI = Confidence interval.

After stratification of the statistical data of subgroups according to PSAD and PI-RADS levels, we observed that with increasing PSAD and PI-RADS, the consistency of diagnosis between mono-TB and TB + SB modes was greater (Figures 3C-D).

### Validity distribution of mono-TB after reasonable stratification

**Figure 4:**
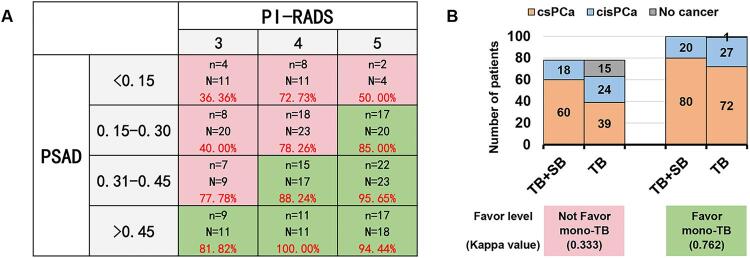
Validity distribution of mono-TB after reasonable stratification. (A) Validity diagnosis rate of mono-TB stratified by combination of PSAD and PI-RADS. The red and green zones represent non-favorable and favorable for mono-TB, respectively. N: number of PCa in this category; n: number of valid diagnoses with mono-TB; Percentage specified in red: valid diagnosis rate of mono-TB. (B) Pathological differences between mono-TB and TB + SB for benign/csPCa/cisPCa detection between non-favorable and favorable mono-TB groups.

Since PSAD and PI-RADS were identified as the main predictors of valid diagnosis with mono-TB, all PCa patients were divided into 12 categories according to PSAD and PI-RADS levels (Figure-4A). Visual increases in PSAD and PI-RADS levels were associated with higher diagnostic validity. Taking the valid diagnostic rate of 80% as the cut-off value, the 12 categories were divided into two zones. The red and green zones represent ‘not favorable’ and ‘favorable’ groups for mono-TB. The columnar distribution comparison chart and weighted kappa test showed that mono-TB and TB + SB results tended to be more consistent for the ‘favorable’ compared to ‘not favorable’ group (0.762 vs. 0.333) (Figure-4B).

TB = Targeted biopsy; PSAD = Prostate-specific antigen density; PI-RADS = Prostate imaging-reporting and data system; PCa = Prostate cancer.

## DISCUSSION

PCa is the leading cancer type in men worldwide. At present, research focus tends to be on treatment of PCa, especially CRPC (13), while prostate biopsy as the only means of initial diagnosis is gradually ignored. Early, large high-quality studies have attempted to determine the optimal biopsy method; that is, TB, SB, or a combination of the two (4, 14, 15). However, ambiguous, and paradoxical conclusions have been obtained. Selection of TB leads to high detection of csPCa, but accurate evaluation of cancer is not achieved, and in some cases, leads to misdiagnosis. Upon selection of SB, the positive rate may be improved to some extent, but the method is associated with inevitable defects of randomness and blindness. Combination of TB and SB has been proposed as the optimal biopsy method but can also lead to oversampling and overdiagnosis. Each biopsy mode has its advantages and disadvantages. In an invited commentary, Olivier Rouvière proposed that it may be unrealistic to implement a strict universal biopsy protocol for all populations (16). In the future, MRI findings, in conjunction with other clinical biomarkers, such as PSAD, may be effectively applied to stratify patients into groups that require TB or SB and those for whom biopsy could be avoided.

In this study, PSAD and PI-RADS were identified as the key predictors in evaluating valid diagnosis with mono-TB. Earlier, Washino et al. (17) proposed that the combination of PI-RADS and PSAD could aid in the decision-making process before initiation of prostate biopsy. The group concluded that biopsy may be unnecessary in patients with PI-RADS ≤ 3 and PSAD < 0.15 ng/mL/mL. Boesen and co-workers (18) proposed an optimal strategy involving biopsy performance only in patients with highly suspicious MRI findings (score > 3) or PSAD ≥ 0.15 ng/mL/mL, which reduced the number of biopsies by 41% and overdiagnosis of cisPCa by 45%, while missing csPCa detection by only 5%. A study by Falagario et al. (19) reported that for men with PI-RADS 1-2, PSAD < 0.10 ng/mL/mL had the highest negative predictive value (98.7%), which decreased to 13.2% for men with PI-RADS 3-5. Schoots et al. (20) additionally proposed a biopsy strategy incorporating MRI findings and PSAD based on a summary of data from the literature. However, their results lack prospective validation.

Two studies involving 89 and 97 patients with PI-RADS 5, respectively, suggested that the additional clinical value provided by SB was minimal and could therefore be excluded when performing TB (21, 22). However, in our opinion, this would be a risky step, since in our study, the valid diagnosis rate of mono-TB was only 25% for patients with PSAD < 0.15, even with a PI-RADS score of 5 (1/4). Liu et al. (23) analyzed the added value of SB to TB from the PSA level and recommended a range of 10.0-20.0 ng/mL for combined SB and TB, while no differences were observed between SB and TB in cases with PSA >20.0 ng/mL and PSA < 10.0 ng/mL. Our study does not dismiss the importance of the role of SB. In total, 16 PCa cases were diagnosed with SB but not TB, although nine of the 16 patients were cisPCa. Moreover, 24 patients were diagnosed as cisPCa with TB, which was upgraded to csPCa following SB. Two recent studies have reported similar results. One included 259 men with PI-RADS lesion scores ≥3 who underwent TB+SB. For the TB+SB mode, detection rates of csPCa, cisPCa, and no cancer were 66%, 6%, and 28%, while for the TB mode, detection rates were 53%, 7%, and 40%, respectively (24). Another study retrospectively evaluated 336 biopsy-naive patients with a single suspicious lesion at mpMRI who also underwent TB+SB. In the TB mode, 40 patients presumed to be negative were actually diagnosed as PCa following SB, including 20 csPCa and 20 cisPCa. In total, 14 cases were identified as cisPCa with TB but diagnosed as csPCa in the SB mode (25). SB cannot be omitted for all patients for several reasons. First, PCa lesions are multifocal and mono-TB may overlook lesions with the highest degree of malignancy. Second, neither software fusion nor cognitive fusion can achieve complete accuracy, and TB errors could be compensated to some extent by SB. Finally, some PCa themselves are MRI-negative and can only be detected with the aid of SB.

A number of indicators have utility in optimizing the biopsy mode, such as the location and size of MRI lesions. Gomez-Gomez et al. (21) suggested that SB can be safely excluded in patients with anterior lesions. Another study including 863 patients with suspected peripheral lesions and negative transitional zone on MRI also confirmed that the detection rate of csPCa was not affected by whether or not the transitional zone was sampled (26). However, we did not observe significant effects of the number, size, and location of lesions on differences in the csPCa detection ability between mono-TB and TB+SB groups. In addition, PSA levels could be affected by 5**α**-reductase inhibitors, and therefore, caution is required in the evaluation of PSAD (27). Prostate-specific membrane antigen ligand positron emission tomography/computed tomography is the current precision imaging examination system for PCa. Further studies are warranted to determine whether optimizing this imaging examination prior to biopsy could potentially provide a reference for the choice of biopsy mode (28-30).

Our results should be interpreted in the context of a number of limitations. First, data were obtained from a single center, and further large-scale randomized controlled trials are needed to verify these findings. Second, TB using the cognitive fusion mode instead of the software fusion mode may have potential bias of inaccurate biopsy localization. Third, TB followed by SB may cause interference in the work of urologists involved in performing SB, such as bleeding tracks, which will affect the implementation of blinding to an extent.

## CONCLUSIONS

In conclusion, among men who underwent biopsy for suspected PCa on MRI (PI-RADS ≥ 3), PSAD combined with PI-RADS effectively predicted PCa and csPCa, and, more importantly, guided optimal selection of the prostate biopsy mode. Higher PSAD and PI-RADS values reflect greater confidence in implementation of TB only and safely omitting SB.

## APPENDIX

Supplementary Table 1 MRI Parameters.


