# Supplementary material for: Is it necessary for all patients with suspicious lesions undergo systematic biopsy in the era of MRI-TRUS fusion targeted biopsy?
Source: Int Braz J Urol. 2023 Mar 20;49(3):359–71. doi: 10.1590/S1677-5538.IBJU.2023.0060 (PMC10335882; doi:10.1590/S1677-5538.IBJU.2023.0060)
Supplement: Supplementary file 1 [file 1677-6119-ibju-49-03-0359-suppl01.pdf]

APPENDIX

Supplementary Table 1 MRI Parameters.

Parameters	T1WI	T2WI	DWI
Sequence	turbo spin-echo	turbo spin-echo	single-shot echo-planar
Imaging plane	Axial	Axial, coronal, sagittal	Axial
Field of view (mm²)	300×300	240×240, 240×240, 240×240	240×240
Matrix (frequency×phase)	256×320	224×320, 224×320, 256×320	64×92
Voxel size (mm³)	0.8×0.8×4.0	0.8×0.8×4.0, 0.8×0.8×4.0, 0.8×0.8×4.0	2.6×2.6×4.0
Slice/Gap (mm)	4/1	4/1, 4/1, 4/1	4/1
Repetition time (msec)	500	6900, 6900, 6900	5600
Echo time (msec)	9	118, 118, 118	83
Flip angle (degrees)	160°	160°, 160°, 160°	90°
b values (s/mm2)	NA	NA-	50/2000
Acceleration factor	2	2	2
Acquisition time (min: s)	1:01	1:57, 1:57, 1:57	3:38

MRI = Magnetic resonance imaging; T1WI = T1-weighted image; T2WI = T2-weighted image; DWI = Diffusion-weighted imaging.

Supplementary Table - 2 Univariate and multivariate Logistic regression analyses to detect PCa or csPCa for TB + SB.

	Detection of PCa				Detection of csPCa			
	Univariate analysis		Multivariate analysis		Univariate analysis		Multivariate analysis	
Variable	OR (95% CI)	P-value	OR (95% CI)	P-value	OR (95% CI)	P-value	OR (95% CI)	P-value
Age (Years)	1.352 (1.088-1.681)	0.007	1.379 (1.077-1.765)	0.011	1.276 (1.024-1.590)	0.030	1.268 (0.993-1.621)	0.057
DRE	1.352 (0.882-2.072)	0.166			1.709 (1.104-2.646)	0.016	1.437 (0.883-2.337)	0.144
PSA (ng/mL)	1.625 (1.316-2.006)	0.001	1.320 (0.894-1.949)	0.162	1.525 (1.232-1.887)	0.001	1.133 (0.769-1.669)	0.528
Prostate-Vol (mL)	0.631 (0.514-0.774)	0.001	0.721 (0.495-1.051)	0.089	0.675 (0.549-0.829)	0.001	0.868 (0.592-1.271)	0.466
PSAD (ng/mL2)	2.302 (1.805-2.937)	0.001	1.516 (0.955-2.407)	0.077	2.053 (1.632-2.584)	0.001	1.635 (1.031-2.592)	0.037
Lesions number	1.073 (0.873-1.317)	0.504			1.179 (0.957-1.452)	0.121		
Lesion size (cm)	2.026 (1.510-2.717)	0.001	1.034 (0.650-1.643)	0.889	2.115 (1.554-2.880)	0.001	1.228 (0.774-1.948)	0.383
Lesion location	1.093 (0.814-1.467)	0.554			0.951 (0.705-1.282)	0.740		
PI-RADS	2.293 (1.722-3.054)	0.001	1.915 (1.222-2.999)	0.005	2.197 (1.657-2.914)	0.001	1.623 (1.063-2.477)	0.025

TB = Targeted biopsy; SB = Systematic biopsy; PCa = Prostate cancer; csPCa = clinically significant prostate cancer;DRE = Digital rectal examination; PSA = Prostate-specific antigen; PSAD = Prostate-specific antigen density; PI-RADS = Prostate imaging-reporting and data system; OR = Odds ratio; CI = Confidence interval.

Supplementary Table 3 - Univariate and multivariate Logistic regression analyses to detect PCa or csPCa for TB.

	Detection of PCa				Detection of csPCa			
	Univariate analysis		Multivariate analysis		Univariate analysis		Multivariate analysis	
Variable	OR (95% CI)	P-value	OR (95% CI)	P-value	OR (95% CI)	P-value	OR (95% CI)	P-value
Age (Years)	1.250 (1.008-1.551)	0.043	1.258 (0.975-1.624)	0.078	1.315 (1.042-1.659)	0.021	1.343 (1.026-1.758)	0.032
DRE	1.655 (1.078-2.543)	0.021	1.369 (0.824-2.274)	0.226	2.258 (1.413-3.609)	0.001	1.995 (1.169-3.403)	0.011
PSA (ng/mL)	1.723 (1.390-2.135)	0.001	1.266 (0.842-1.904)	0.258	1.633 (1.298-2.055)	0.001	1.099 (0.718-1.683)	0.664
Prostate-Vol (mL)	0.601 (0.488-0.739)	0.001	0.759 (0.510-1.129)	0.174	0.656 (0.526-0.818)	0.001	0.935 (0.609-1.437)	0.760
PSAD (ng/mL^2)	2.658 (2.062-3.425)	0.001	1.855 (1.143-3.009)	0.012	2.243 (1.760-2.858)	0.001	1.952 (1.172-3.251)	0.010
Lesions number	1.075 (0.876-1.320)	0.490			1.021 (0.821-1.269)	0.854		
Lesion size (cm)	2.230 (1.648-3.018)	0.001	1.081 (0.667-1.752)	0.751	2.582 (1.821-3.661)	0.001	1.400 (0.838-2.338)	0.199
Lesion location	1.096 (0.816-1.471)	0.543			1.234 (0.901-1.689)	0.191		
PI-RADS	2.546 (1.905-3.403)	0.001	2.040 (1.290-3.225)	0.002	2.611 (1.929-3.535)	0.001	1.815 (1.162-2.837)	0.009

TB = Targeted biopsy; PCa = Prostate cancer; csPCa = clinically significant prostate cancer;DRE = Digital rectal examination; PSA = Prostate-specific antigen; PSAD = Prostate-specific antigen density; PI-RADS = Prostate imaging-reporting and data system; OR = Odds ratio; CI = Confidence interval.
